# Metformin and tenovin‐6 synergistically induces apoptosis through LKB1‐independent SIRT1 down‐regulation in non‐small cell lung cancer cells

**DOI:** 10.1111/jcmm.14194

**Published:** 2019-02-01

**Authors:** Bo Bin Lee, Yujin Kim, Dongho Kim, Eun Yoon Cho, Joungho Han, Hong Kwan Kim, Young Mog Shim, Duk‐Hwan Kim

**Affiliations:** ^1^ Department of Molecular Cell Biology Samsung Biomedical Research Institute, Sungkyunkwan University School of Medicine Suwon Korea; ^2^ Department of Pathology Samsung Medical Center, Sungkyunkwan University School of Medicine Seoul Korea; ^3^ Department of Thoracic and Cardiovascular Surgery Samsung Medical Center, Sungkyunkwan University School of Medicine Seoul Korea

**Keywords:** LKB1, metformin, non‐small cell lung cancer (NSCLC), SIRT1 inhibitor, tenovin‐6

## Abstract

Sirtuin 1 (SIRT1) is known to play a role in a variety of tumorigenesis processes by deacetylating histone and non‐histone proteins; however, antitumour effects by suppressing SIRT1 activity in non‐small cell lung cancer (NSCLC) remain unclear. This study was designed to scrutinize clinicopathological significance of SIRT1 in NSCLC and investigate effects of metformin on SIRT1 inhibition. This study also evaluated new possibilities of drug combination using a SIRT1 inhibitor, tenovin‐6, in NSCLC cell lines. It was found that SIRT1 was overexpressed in 300 (62%) of 485 formalin‐fixed paraffin‐embedded NSCLC tissues. Its overexpression was significantly associated with reduced overall survival and poor recurrence‐free survival after adjusted for histology and pathologic stage. Thus, suppression of SIRT1 expression may be a reasonable therapeutic strategy for NSCLC. Metformin in combination with tenovin‐6 was found to be more effective in inhibiting cell growth than either agent alone in NSCLC cell lines with different liver kinase B1 (LKB1) status. In addition, metformin and tenovin‐6 synergistically suppressed SIRT1 expression in NSCLC cells regardless of LKB1 status. The marked reduction in SIRT1 expression by combination of metformin and tenovin‐6 increased acetylation of p53 at lysine 382 and enhanced p53 stability in LKB1‐deficient A549 cells. The combination suppressed SIRT1 promoter activity more effectively than either agent alone by up‐regulating hypermethylation in cancer 1 (HIC1) binding at SIRT1 promoter. Also, suppressed SIRT1 expression by the combination synergistically induced caspase‐3‐dependent apoptosis. The study concluded that metformin with tenovin‐6 may enhance antitumour effects through LKB1‐independent SIRT1 down‐regulation in NSCLC cells.

## 1 INTRODUCTION

Lung cancer is the most common cause of cancer‐related death in the world. Despite significant advances in its diagnosis and treatment, its prognosis remains extremely poor.^1^ Currently, a number of agents targeting various molecular pathways are under development or being used in lung cancer treatment. Molecular therapies targeting epithelial growth factor receptor (EGFR),^2^ vascular endothelial growth factor (VEGF),^3^ and echinoderm microtubule associated protein‐like 4 (EML4) and anaplastic lymphoma kinase (ALK) fusion oncogene^4^ have been demonstrated to possess significant efficacies against lung cancer.^5^ However, failure to achieve long‐lasting efficacy with a single agent has been observed because cancer cells can acquire resistance during long‐term treatment with a single agent such as EGFR‐tyrosine kinase inhibitor (TKI) or ALK inhibitor.^6^, ^7^ Therefore, this study designed a combination treatment with a new therapeutic target to achieve more effective response than single‐agent lung cancer treatment. However, combination therapy using a therapeutic dosage of each individual drug is generally more toxic than single‐agent therapy.^8^, ^9^ To overcome this problem, this study asked whether a combination treatment at lower concentrations instead of concentrations of each single agent commonly used in vitro could have synergistic effects.

Metformin is an oral antidiabetic drug used to treat type II diabetes. It is also being tested as an anticancer agent because of its ability to suppress cancer growth in vitro and in vivo.^10^, ^11^, ^12^, ^13^, ^14^ Metformin is well‐known to regulate cell growth through inhibition of mammalian target of rapamycin complex 1 (mTORC1) signalling pathway by activating the AMP‐activated protein kinase (AMPK).^15^, ^16^ AMPK, a highly conserved intracellular energy sensor and modulator of cell growth, is activated upon decline in adenosine triphosphate (ATP). AMPK is activated by serine/threonine kinase LKB1, a major kinase phosphorylating AMPK under conditions of energy stress.^17^ Metformin is known to trigger its activation through LKB1‐dependent phosphorylation of AMPK under conditions of low intracellular ATP.^18^ LKB1 is inactivated by somatic mutation in approximately 30% of NSCLCs.^19^ However, the molecular mechanism involved in the antitumour effect of metformin that is dependent on LKB1 status remains unclear in NSCLC cells. Recent studies have indicated that metformin may sensitize cancer cells to chemotherapy agents in lung cancer.^20^, ^21^ For example, metformin and EGFR‐TKI have a synergistic effect in treating NSCLC patients with diabetes mellitus type 2.^22^ Moreover, metformin can reverse crizotinib resistance by inhibiting type I insulin‐like growth factor receptor (IGF‐1R) signalling in crizotinib‐resistant human lung cancer cells.^23^ Metformin and sorafenib can synergistically inhibit tumour growth by activating the AMPK pathway in NSCLC cells both in vitro and in vivo*.* 24 Thus, combination of metformin with other chemotherapy agents may improve treatment outcome for NSCLC patients.

SIRT1, also known as NAD^+^‐dependent deacetylase sirtuin‐1, is a homolog of the silent information regulator 2 (*Sir2*) gene in yeast. It is involved in diverse cellular processes including metabolism, senescence and tumour initiation and progression, by modulating the deacetylation of histone and non‐histone proteins.^25^, ^26^ SIRT1 is overexpressed in several human cancers. It is known to play a role in cancer drug resistance by modulating several targets and in the activation of AMPK.^27^, ^28^, ^29^ SIRT1 mainly regulates various transcription factors such as tumour suppressor p53, forkhead box protein O1 (FOXO1) and forkhead box class O 3a (FOXO3a) of forkhead transcription factors, peroxisome proliferator‐activated receptor‐γ coactivator (PGC)‐1α, histone acetyltransferase p300 and nuclear factor kappa B (NFkB) in the nucleus.^29^, ^30^ Thus, inhibition of SIRT1 expression could have promising therapeutic potential for NSCLC. This study examines the hypothesis that SIRT1 may be an important target for metformin.

HIC1 is an epigenetically regulated sequence‐specific transcriptional repressor in many cancers including prostate, pancreatic and oesophageal cancers.^31^, ^32^, ^33^ Inactivation of HIC1 expression is known to up‐regulate SIRT1 expression and allow cells to bypass apoptotic cell death.^34^, ^35^ HIC1 is also known to play a critical role in DNA damage response.^36^, ^37^ HIC1 forms a transcriptional repression complex with SIRT1 through an N‐terminal POZ (Pox virus and zinc finger) domain. This complex controls SIRT1 expression by directly binding to the SIRT1 promoter.^38^ The SIRT1 promoter has three HIC1 binding sites at −1116, −1039 and −8 bp regions from the transcription start site (NCBI Refseq: NT_030059.14).^39^, ^40^ HIC complexes can differentially bind on two mutually exclusive HIC1 binding sites (distal site and proximal site) on the SIRT1 promoter.^41^ Occupancy of distal sites by HIC1 complex was regulated by serum starvation time. Although the mechanism by which HIC affects SIRT1 down‐regulation has been explored, little is known about the mechanism involved in the regulation of anticancer activity of metformin in NSCLC cells by SIRT1.

Tenovin‐6 is a small‐molecule inhibitor of both SIRT1 and SIRT2 that can inhibit cell growth in various cancer types.^42^, ^43^ Tenovin‐6 is known to enhance cytotoxic effects of 5‐fluorouracil and oxaliplatin in colon cancer cells.^44^ It has shown very encouraging in vivo effects against cancers in animal experiments.^45^, ^46^ Moreover, tenovin‐6 is more water‐soluble than tenovin‐1.^45^ Tenovin‐6 can inhibit protein deacetylating activities of SIRT1 and SIRT2 and promotes p53 acetylation in cancer cells.^47^, ^48^ Although its effect is limited owing to its low specificity. It also induces apoptosis and results in dysregulated autophagy.^49^ However, these inhibitors are not considered sufficiently potent to improve patient prognosis. Therapeutic application of SIRT1 inhibitors needs to be investigated in combination with other agents. Therefore, this study determined whether tenovin‐6 might be suitable for administration to cancer cells together with metformin because of its potent anticancer effects and water solubility.

The objective of this study was to analyse clinicopathological significance of SIRT1 overexpression using 485 formalin‐fixed paraffin‐embedded NSCLC tissues. In addition, this study investigated a possible molecular mechanism of the anticancer effect of metformin plus SIRT1 inhibitor, tenovin‐6 in NSCLC cells irrespective of LKB1 status.

## 2 MATERIALS AND METHODS

### 2.1 Study population

This study obtained a total of 485 formalin‐fixed paraffin‐embedded tissues from NSCLC patients who were undergoing surgical resection between May 1994 and April 2004 at Samsung Medical Center in Seoul, Korea. Informed consent was obtained from each patient before surgery. This study was approved by the Institutional Review Board of Samsung Medical Center. Post‐operative follow‐up to detect recurrence was performed as previously described.^50^ Follow‐up data were available until November 2016. NSCLC was staged according to the guidelines of the tumour‐node‐metastasis (TNM) classification of the American Joint Committee on Cancer.^51^

### 2.2 Immunohistochemistry

The construction of tissue microarrays (TMAs) from paraffin blocks prepared from the NSCLC samples and immunohistochemical staining of SIRT1 were performed as previously described.^52^ A rabbit anti‐human SIRT1 polyclonal antibody (Santa Cruz Biotechnology) was used as the primary antibody. All available slides were evaluated in a blinded fashion by two authors (EY Cho and D‐H Kim) to reduce interobserver variability. SIRT1 was considered to be overexpressed if immunoreactivity was found in at least 10% of all nuclei. Expression levels of SIRT1 protein were calculated by multiplying the intensity score (0, none; 1, weak; 2, moderate; 3, strong) and the proportion score of positive staining tumour cells (0, absent; 1, 0% to 10%; 2, 10% to 50%; 3, 50% to 80%; 4, >80%). The cut‐off value for overexpression was determined by comparison with an internal control consisting of 32 normal lung cores.

### 2.3 Cell lines and reagents

A549 and H460 NSCLC cells (LKB1 negative),^53^ and H1299, H1650 and H226 NSCLC cells (LKB1 positive)^54^, ^55^, ^56^ were purchased from the American Type Culture Collection (Rockville, MD, USA). H1299, H226 and H460 cells were cultured in complete RPMI 1640 medium supplemented with 10% heat‐inactivated foetal bovine serum (FBS) (GIBCO‐BRL, Grand Island, NY, USA) in a humidified incubator at 37°C with 5% CO_2_. A549 cells were cultured in RPMI 1640 medium with 10 mmol/L HEPES. Metformin (Sigma‐Aldrich, St. Louis, MO, USA) and tenovin‐6 (Cayman Chemical, Ann Arbor, MI, USA) were dissolved in water and dimethyl sulphoxide (DMSO) respectively. They were diluted with phosphate‐buffered saline (PBS). The final concentration of DMSO did not exceed 0.1% (v/v). Cycloheximide (CHX) solution (Sigma‐Aldrich) was used in CHX chase assay to determine protein stability.

### 2.4 Cell viability assay

Cells were seeded into six‐well plates at a density of 2  ×  10^5^ cells/mL and then treated with metformin and/or tenovin‐6 for 48 hours. After treatment, cells were harvested by trypsinization and stained with 0.4% trypan blue solution (GIBCO‐BRL). The number of viable cells was counted using a haemocytometer, and cell viability was expressed as the percentage of live cells. IC_50_ values were determined using CellTiter 96^®^ AQueous One Solution Cell Proliferation Assay (MTS, 3‐(4,5‐dimethylthiazol‐2‐yl)‐5‐(3‐carboxymethoxyphenyl)‐2‐(4‐sulphophenyl)‐2*H*‐tetrazolium inner salt) (Promega, Madison, WI, USA) according to the manufacturer's protocol.

### 2.5 Soft agar colony formation assay

After A549 cells were treated with metformin and/or tenovin‐6 for 48 hours, cells were trypsinized. Cell suspension was mixed with 0.3% soft agar in growth medium and layered (1000 cells/well in 6‐well plates) on top of 0.6% base agar with growth medium. After 2 weeks, cells were stained with a nitro blue tetrazolium chloride (Sigma‐Aldrich) solution (1 mg/mL in PBS) overnight at 37°C. Colonies containing more than 50 individual cells and those with diameter greater than 0.5 μm were counted using Image J software (National Institutes of Health, Bethesda, MD, USA). All experiments were performed in triplicate.

### 2.6 Quantitative real‐time RT‐PCR

Total RNA was extracted from cultured cells using an RNeasy Mini Kit (Qiagen, Valencia, CA, USA) and cDNA was synthesized using a QuantiTect Reverse Transcription Kit (Qiagen). After cDNA synthesis, a quantitative real‐time PCR was performed with an ABI PRISM 7900HT system (Applied Biosystems, Foster City, CA, USA) and SYBR Green PCR Master Mix (Applied Biosystems) according to the manufacturer's protocol. Primers sequences are listed in Table 1.

**Table 1:** Primer sequences used in RT‐qPCR

Target gene	Forward (5′‐3′)	Reverse (5′‐3′)
SIRT1	CCTGACTTCAGATCAAGAGACGGT	CTGATTAAAAATGTCTCCACGAACAG
HIC1	GTCGTGCGACAAGAGCTACAA	CGTTGCTGTGCGAACTTGC
APAF1	GCAGCCAGCTTCAGGATCTAC	CAAAGTTCCTTGTGCATCTTGG
BAX	GGACGAACTGGACAGTAACATGG	GCAAAGTAGAAAAGGGCGACAAC
BAK1	ATGGTCACCTTACCTCTGCAA	TCATAGCGTCGGTTGATGTCG
NOXA	ACTGTTCGTGTTCAGCTC	GTAGCACACTCGACTTCC
PUMA	ACCTCAACGCACAGTACGAG	CCCATGATGAGATTGTACAGGA
DR5	GCCCCACAACAAAAGAGGTC	GGAGGTCATTCCAGTGAGTG
DDIT3	AGCAGAGGTCACAAGCACCT	CTGGGGAATGACCACTCTGT
GADD45A	AACGGTGATGGCATCTGAAT	CCCTTGGCATCAGTTTCTGT
TNFRSF10A	GGATGGTCAAGGTCAAGGATT	CAGCAACGGAACAACCAAAG

### 2.7 Immunoblot analysis

Cultured cells were lysed in buffer containing 20 mmol/L Tris‐HCl (pH 7.5), 150 mmol/L NaCl, 1 mmol/L Na_2_EDTA, 1 mmol/L EGTA, 1% NP‐40, 1% sodium deoxycholate, 2.5 mmol/L sodium pyrophosphate, 1 mmol/L β‐glycerophosphate, 1 mmol/L Na_3_VO_4_, and 1 µg/mL leupeptin. The buffer was supplemented with 1 mmol/L PMSF immediately before cell lysis. Lysates were sonicated and centrifuged at 13 000 rpm for 15 minutes at 4°C. Samples were subjected to 8% or 12% sodium dodecyl sulphate‐polyacrylamide gel electrophoresis (SDS‐PAGE) followed by transfer to polyvinylidene difluoride membrane (Millipore, Bedford, MA, USA). After membranes were blocked with 5% non‐fat dry milk (Bio‐Rad, Hercules, CA, USA) in Tris‐buffered saline with 0.1% Tween 20 (TBS‐T) at room temperature for 1 hour, they were then incubated with anti‐SIRT1, acetyl‐p53 (K382), cleaved PARP, PARP, cleaved caspase‐3, caspase‐3 antibody (Cell Signaling Technology, Beverly, MA, USA), anti‐LKB1, p21, GADD45α antibody (Santa Cruz Biotechnology, Santa Cruz, CA, USA), anti‐p53 antibody (Invitrogen, Carlsbad, CA, USA), anti‐Flag‐M2, α‐tubulin or β‐actin antibody (Sigma‐Aldrich) at 4°C overnight. After washing with TBS‐T, membranes were incubated with horseradish peroxidase (HRP)‐conjugated secondary antibody at room temperature for 2 hours. Protein bands were visualized using a SuperSignal West Pico Chemiluminescent Substrate (Invitrogen).

### 2.8 Immunofluorescence

After cells were grown on glass coverslips and treated with 10 mmol/L metformin, cells were then fixed with 4% paraformaldehyde diluted in PBS at indicated time points. For immunostaining, cells were blocked with PBS containing 5% normal goat serum and 0.3% Triton X‐100 for 60 min. They were then incubated with antibodies against acetyl‐p53 (K382) (Abcam, Cambridge, UK) diluted in PBS containing 1% BSA and 0.3% Triton X‐100 at 4°C overnight. After washing three times with PBS, slides were incubated with secondary antibodies (AlexaFluor488 goat anti‐rabbit, Invitrogen) at room temperature for 1 hour. Slides were then washed with PBS, counterstained with ProLong^®^ gold antifade reagent (No. P36930, Invitrogen) with 4′,6‐diamidino‐2‐phenylindole (DAPI, Cell Signaling Technology) and analysed by confocal microscopy.

### 2.9 Chromatin immunoprecipitation assay

The chromatin immunoprecipitation (ChIP) assay was performed with an EZ‐ChIP kit (Millipore) and salmon sperm DNA/protein A agarose (Millipore) according to the manufacturer's instructions. A549 cells were cultured in RPMI1640 medium containing 10 mmol/L metformin and/or 10 µmol/L tenovin‐6 for 48 hours. Cells were cross‐linked with 1% formaldehyde (Sigma‐Aldrich) for 10 minutes at 37°C and lysed in SDS lysis buffer. Lysates were then sonicated to shear cross‐linked DNA to fragments of 200 to 1000 base pairs in length. These DNA fragments were immunoprecipitated with an antibody against HIC1 or normal rabbit IgG (Santa Cruz Biotechnology). Purified DNA was then subjected to PCR and qPCR. Primer sequences used to amplify three HIC1 binding sites in the SIRT1 promoter region are shown in Table 2.

**Table 2:** Primer sequences used in ChIP

Primer name	Sequence (5′‐3′)
−1143 to −859 on SIRT1 (PCR)	F:GATAGAAACGCTGTGCTCCA
	R:CCTTCCTTTCTAGCGTGAGC
−8 on SIRT1 (PCR & qPCR)	F:GGTCACGTGATGGGGTTTA
	R:CCATCTTCCAACTGCCTCTC
−1116 on SIRT1 (qPCR)	F:TAGAAACGCTGTGCTCCAGG
	R:AGGACCCATATAACCCATGGTAGA
−1039 on SIRT1 (qPCR)	F:TCTACCATGGGTTATATGGGTCCT
	R:GGAAAGCCCTTCCACTTTCCT

### 2.10 Luciferase reporter assay

SIRT1 promoter plasmid (pSIRT1‐Gluc) containing Gaussia Luciferase (GLuc) as a reporter (vector pEZX‐PG02) was purchased from GeneCopoeia (Rockville, MD, USA). This pSIRT1‐Gluc plasmid was cotransfected into A549 cells with a wild‐type HIC1 expression construct. After transfection, cells were treated with 10 mmol/L metformin and/or 5 µmol/L tenovin‐6. Luciferase activity was measured using a Gaussia luciferase assay kit (Promega) according to the manufacturer's instructions.

### 2.11 Apoptosis assay

A549 cells were treated with metformin in the presence or absence of tenovin‐6 for up to 48 hours to measure apoptosis. Apoptosis was analysed by measuring relative expression levels of pro‐apoptotic genes such as apoptotic protease activating factor 1 (APAF1), Bcl‐2 homologous antagonist/killer (BAK1), BCL2‐associated X protein (BAX), DNA damage‐inducible transcript 3 (DDIT3), death receptor 5 (DR5), growth arrest and DNA‐damage‐inducible protein GADD45 alpha (GADD45A), NOXA, p53 up‐regulated modulator of apoptosis (PUMA) and tumour necrosis factor receptor superfamily member 10a (TNFRSF10A) using annexin V‐FITC/propodium iodide (PI) staining and TUNEL assays. Cells were stained with an annexin V‐FITC apoptosis detection kit I (BD Biosciences, San Jose, CA, USA). Data were analysed with a FACs Calibur flow cytometer using CellQuest PRO software (BD Biosciences). Apoptosis was also analysed using DeadEnd™ Fluorometric TUNEL System (Promega) according to the manufacturer's instructions. Apoptotic cells were detected by confocal microscopy. Immunoblot analysis was also performed to detect activated caspase‐3 and poly‐ADP‐ribose polymerase (PARP) cleavage as markers of apoptosis induction. To detect caspase activity, a Caspase‐Glo^®^ 3/7 Assay (Promega) was used according to the manufacturer's instructions.

### 2.12 Statistical analysis

Associations of SIRT1 overexpression with continuous (or categorical) variables were analysed using the *t* test (or Wilcoxon rank‐sum test) or Pearson's chi‐square test (or Fisher's exact test). Multivariate logistic regression analysis was performed to identify independent risk factors affecting SIRT1 overexpression. This study also evaluated the effect of SIRT1 overexpression on patient survival using the Kaplan‐Meier method and compared significant differences in survival between the two groups by the log‐rank test. Cox proportional hazards regression analysis was performed to estimate hazard ratios of independent prognostic factors for survival, after adjusting for potential confounders. All statistical analyses were two‐sided with a type I error rate of 5%.

## 3 RESULTS

### 3.1 SIRT1 overexpression correlates with poor overall and recurrence‐free survival in NSCLC patients

**Figure 1:**
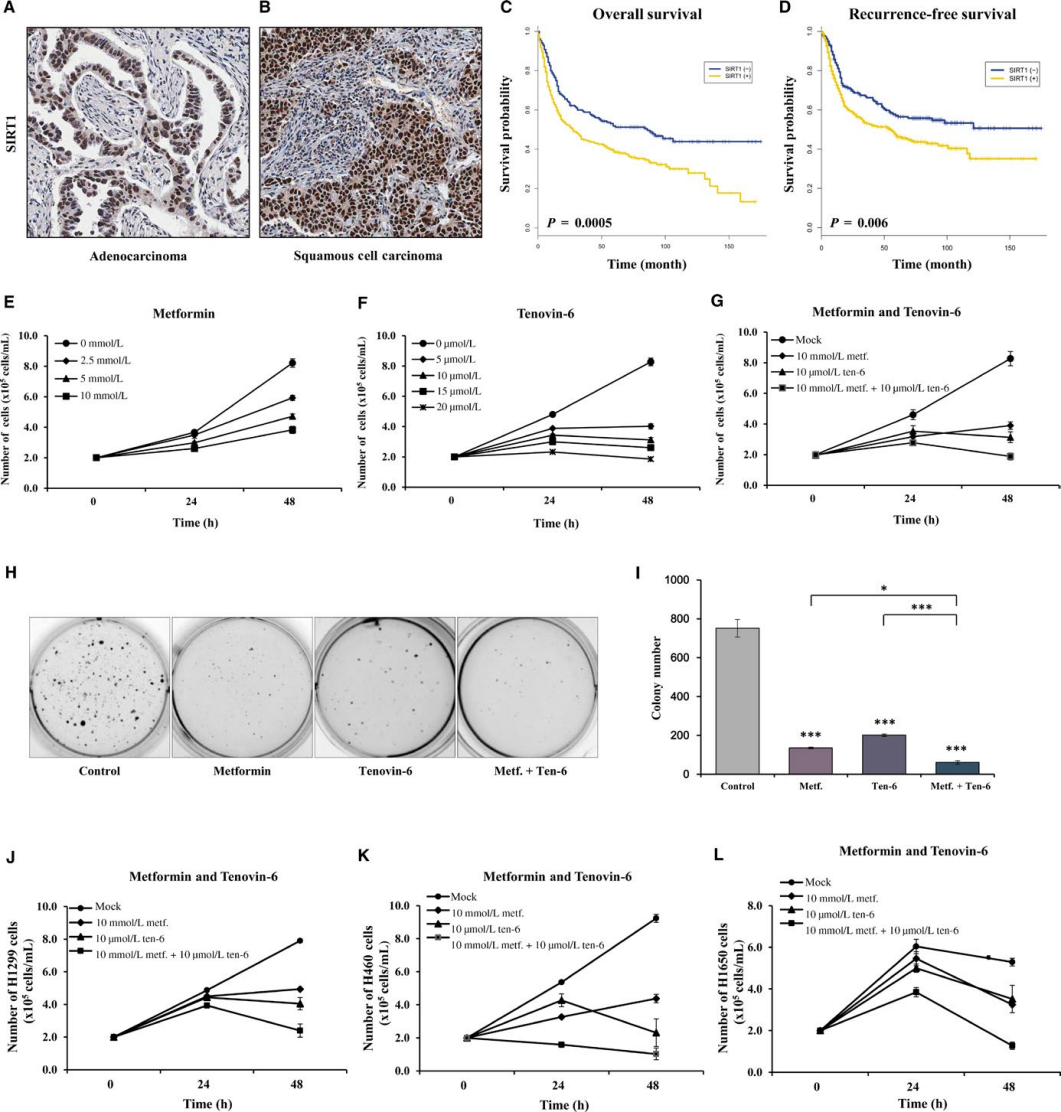
Overexpression of sirtuin 1 (SIRT1) in non‐small cell lung cancers (NSCLCs) and effects of metformin and tenovin‐6 on cell growth in NSCLC cell line A549 (A&B) Immunohistochemical staining of SIRT1 was performed for 485 formalin‐fixed paraffin‐embedded tissues. Representative positive staining is shown in the nuclei of adenocarcinoma (A) and squamous cell carcinoma (B) cells. (×200). (C and D) Overall survival (C) and recurrence‐free survival (D) were compared between patients with and without SIRT1 overexpression using Kaplan‐Meier survival curves. *P*‐values were based on the log‐rank test. (E‐G) A549 cells were treated with metformin and tenovin‐6 either alone or in combination for the indicated time, and cell viability was determined by trypan blue assay. Each experiment was carried out in triplicate. Error bars indicate standard deviation (SD). (H and I) Colony formation assay was performed after treatment with 10 mmol/L metformin, alone or in combination with 10 μmol/L tenovin‐6, for 48 h in A549 cells and quantitated. Error bars indicate mean ± SD; *P < 0.05, ***P < 0.001 (Student's *t* test). Results shown are representative of three independent experiments. (J‐L) H1299 (wtLKB1), H460 (mtLKB1) and H1650 (wtLKB1) cells were treated with 10 mmol/L metformin and 10 μmol/L tenovin‐6 alone or in combination for 48 h. Cell viability was determined by the trypan blue assay. Results are shown as mean ±SD

This study analysed the association of SIRT1 overexpression with continuous and categorical variables in NSCLC patients. Clinicopathological characteristics of the 485 participants are described in Table 3. Positive staining for SIRT1 protein is shown in Figure 1A,B. It was overexpressed in 300 (62%) of 485 patients. SIRT1 overexpression was not associated with patient age, pathologic stage or exposure to tobacco smoke. However, overexpression did occur more frequently in adenocarcinoma than in squamous cell carcinoma (68% vs 54%, *P* = 0.004). Patients with SIRT1 overexpression showed significantly reduced overall survival (*P* = 0.0005; Figure 1C) and poor recurrence‐free survival (RFS; *P* = 0.006; Figure 1D). The median follow‐up duration was 62 months. Five‐year RFS rates in patients with SIRT1 overexpression and those without SIRT1 overexpression were 57% and 46% respectively. Overall survival in patients with SIRT1 overexpression was 1.54 times (95% confidence interval [CI] = 1.21 − 1.97, *P* = 0.0006) poorer than that in those without SIRT1 overexpression after adjusting for pathologic stage, age, histology, and recurrence (Table 4). SIRT1 overexpression was also associated with poor RFS (adjusted hazard ratio [HR] = 1.44, 95% CI = 1.09 − 1.91, *P* = 0.01).

**Table 3:** Clinicopathological characteristics of the study participants (N = 485)

Variables	SIRT1		*P*
	Normal (185)	Overexpression (N = 300)	
Age	60 ± 12	61 ± 9	0.27
Pack‐years (smoking)	31 ± 26	29 ± 27	0.44
Size (cm)	4.2 ± 2.1	4.1 ± 2.1	0.60
Sex			
Male	142	228	
Female	43	72	0.85
Smoking status			
Never	40	79	
Former	22	31	
Current	86	129	0.45
Histology			
Adeno	68	144	
Squamous	105	124	
Others	12	32	0.004
Pathologic stage			
I	82	133	
II	64	94	
III	35	70	
IV	0	1	0.62
Differentiation			
Well	31	46	
Moderately	87	131	
Poorly	28	51	
Undifferentiated	0	6	0.24
Recurrence			
No	108	153	
Yes	77	147	0.11

Adeno, adenocarcinoma; Squamous, squamous cell carcinoma.

**Table 4:** Cox proportional hazards analysis of survival

	SIRT1 overexpression	HR	95% CI	*P*
Overall survival^a^	No	1.00		
	Yes	1.54	1.21‐1.97	0.0006
RFS^b^	No	1.00		
	Yes	1.44	1.09‐1.91	0.01

CI, confidence interval; HR, hazard ratio; RFS, recurrence‐free survival.

a Adjusted for age, recurrence and pathologic stage.

b Adjusted for histology and pathologic stage.

### 3.2 Metformin and tenovin‐6 synergistically inhibit cell growth in NSCLC cells

This study showed that SIRT1 overexpression was associated with poor overall and recurrence‐free survival in NSCLC. Thus, whether SIRT1 inhibitor tenovin‐6 could enhance the anticancer effect of metformin by inhibiting SIRT overexpression in NSCLC cells was determined. First, this study compared effects of metformin‐induced growth inhibition as a single agent and in combination with tenovin‐6 in NSCLC cells. Concentrations of metformin and tenovin‐6 used in this study were based on the MTS assay. IC_50_ values for metformin and tenovin‐6 in functionally LKB1‐negative A549 cells were 28.7 mmol/L and 21.1 μmol/L respectively (data not shown). However, this study used lower concentrations of metformin and tenovin‐6 because high doses of metformin in vitro were controversial in clinical application.^57^, ^58^, ^59^

Metformin (Figure 1E) and tenovin‐6 (Figure 1F) inhibited A549 cell proliferation in time‐ and dose‐dependent manners. Metformin at 10 mmol/L (<half of its IC_50_) and tenovin‐6 at 10 μmol/L (<half of IC_50_) in combination inhibited the proliferation more effectively than either monotherapy alone (Figure 1G). To test the combination effect, CDI (coefficient of drug interaction) was calculated after 48 hours treatment with metformin and tenovin‐6. Results are shown in Figure 1G. CDI was calculated according to the following equation: CDI  =  AB/(A × B) (AB, relative cell viability of the combination; A or B, relative cell viability of the single agent groups).^60^ Usually, CDI < 1 indicates a synergistic effect. Our data suggested that drug actions were synergistic (CDI = (2.2/8)/[(6/8)(3.8/8)] = 0.772) when 10 mmol/L metformin was combined with 10 μmol/L tenovin‐6. Therefore, the combination of metformin and tenovin‐6 showed synergism in suppressing cell growth. Consistent with this result, colony formation assay using A549 cells showed that the number of cell colonies was significantly decreased in metformin or tenovin‐6 alone group than that in the control (Figure 1H,I). In addition, combined treatment of metformin and tenovin‐6 reduced colonies by 8% of initial plating density compared with control in A549 cells. This study also observed significantly decreased growth of wild‐type LKB1 H1299 and H1650 as well as functionally LKB1‐negative H460 under the same experimental conditions (Figure 1J‐L). These results confirmed that tenovin‐6 sensitized the effect of metformin on controlling NSCLC cell growth irrespective of LKB1.

### 3.3 Metformin and tenovin‐6 synergistically down‐regulate SIRT1 expression in NSCLC cells irrespective of LKB1 status

**Figure 2:**
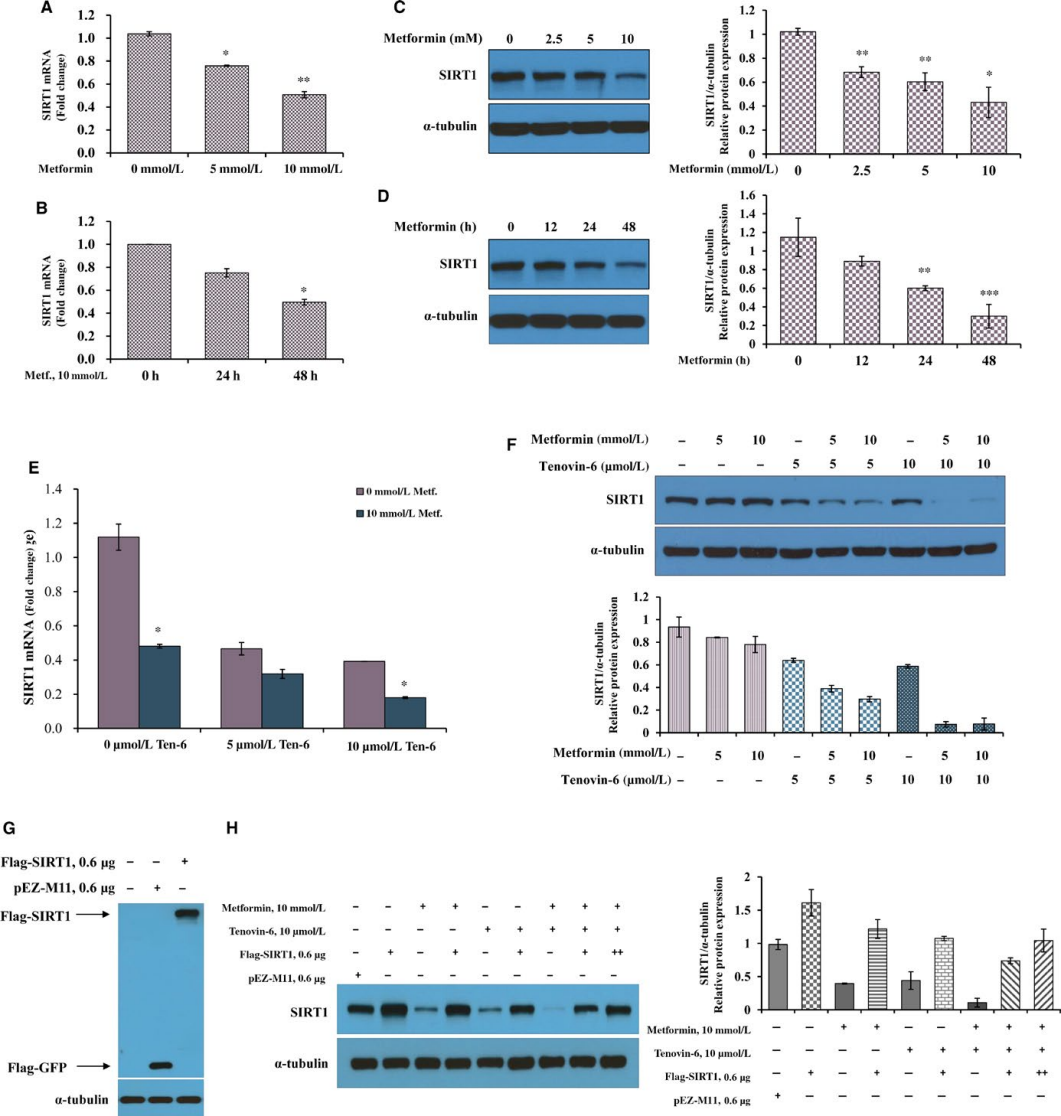
Metformin and tenovin‐6 synergistically suppress sirtuin 1 (SIRT1) expression. (A and C) A549 cells were treated with indicated concentrations of metformin for 48 h. mRNA (A) and protein (C) levels of SIRT1 were analysed by quantitative real‐time RT‐PCR (qRT‐PCR) and immunoblotting, respectively. (B and D) A549 cells were treated with 10 mmol/L metformin for the indicated time, and SIRT1 mRNA (B) and protein (D) levels were detected by qRT‐PCR and immunoblotting, respectively. Results are shown as mean ± SD; *P < 0.05, **P < 0.01, ***P < 0.001. This experiment was performed three times. (E and F) A549 cells were treated with metformin and tenovin‐6 at indicated concentrations for 48 h, and SIRT1 mRNA (E) and protein (F) levels were detected by qRT‐PCR and immunoblotting, respectively. Error bars indicate mean ± SD; *P < 0.05. (G) A549 cells were transfected with Flag‐SIRT1 (Flag‐tagged SIRT1^wt^) or pEZ‐M11 (Flag‐GFP) as a control vector. Immunoblotting were performed using anti‐FLAG antibodies. (H) A549 cells were transfected with the control vector or Flag‐SIRT1 expression vector. Four hours post‐transfection, the cells were treated with 10 mmol/L metformin or 10 μmol/L tenovin‐6 for 48 h, and SIRT1 expression was analysed using immunoblotting in cell lysates. Bar graphs represent mean ± SD of SIRT1/α‐tubulin from three independent experiments

This study explored whether the antiproliferative effect of the combination of metformin with tenovin‐6 was mediated by SIRT1 expression. Whether metformin regulated SIRT1 expression by metformin in functionally LKB1‐deficient A549 cells was first investigated. SIRT1 mRNA (Figure 2A,B) and protein expression levels (Figure 2C,D) in A549 cells treated with metformin were decreased in dose‐ and time‐dependent manner. This study also asked whether the combination of metformin with tenovin‐6 would have synergistic effects to regulate SIRT1 expression. Combined treatment resulted in more significant suppression of mRNA and protein levels of SIRT1 than treatment with metformin alone (Figure 2E,F). To determine whether metformin and tenovin‐6 directly regulated SIRT1 expression, ectopic expression of Flag‐tagged SIRT1^wt^ was performed (Figure 2G,H). SIRT1 overexpression was confirmed by immunoblot analysis (Figure 2H). Metformin or tenovin‐6 reduced SIRT1 levels (lane 3 or lane 5 vs lane 1). Following ectopic expression of SIRT1, treating cells with metformin (lane 4 vs lane 3) or tenovin‐6 (lane 6 vs lane 5) rescued SIRT1 levels. In consistent with Figure 2F, metformin with tenovin‐6 synergistically down‐regulated SIRT1 expression (lane 7 vs lane 1). The combination of metformin and tenovin‐6 after ectopic SIRT1 expression resulted in significant reduction in SIRT1 level (lane 8 vs lane 2). There was more SIRT1 reduction in the combined treatment transfected SIRT1wt compared with either monotherapy after ectopic SIRT1 expression (lane 8 vs lane 4 or lane 6). Increasing numbers of constructs that expressed flag‐tagged SIRT1 restored SIRT1 reductions caused by combined treatment with both metformin and tenovin‐6 (lane 9 vs lane 8).

**Figure 3:**
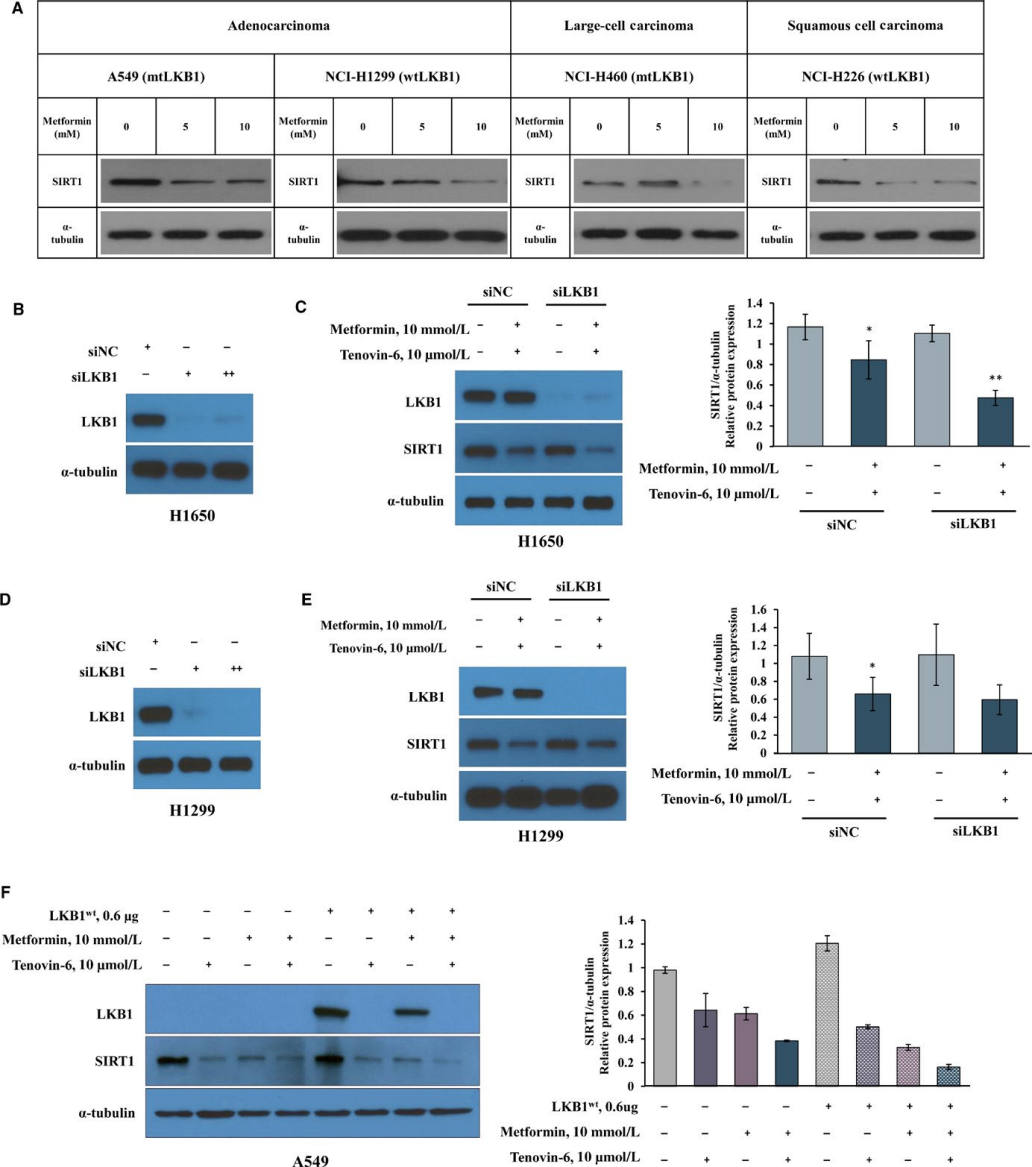
Effect of metformin and tenovin‐6 on sirtuin 1 (SIRT1) expression is not dependent on liver kinase B1 (LKB1). (A) Lung cancer cells with mutant LKB1 (A549 and H460 cell lines) and wild‐type LKB1 (H1299 and H226 cell lines) were treated with indicated concentration of metformin, and SIRT1 protein levels were analysed 48 h later by immunoblotting. (B‐E) H1650 and H1299 (wild‐type LKB1) cell lines were separately transfected by siLKB1 for 48 h. LKB1 was knocked down in H1650 cells (B) and H1299 (D) with specific siRNA. Four hours post‐transfection, cells were incubated with 10 mmol/L metformin and 10 µmol/L tenovin‐6 for 48 h. Expression levels of LKB1 and SIRT1 proteins were determined using immunoblotting in H1650 cells (C) and H1299 cells (E), respectively. (F) Ectopic expression of LBK1 was accomplished in A549 cells using transient transfection. Four hours post‐transfection, A549 cells were incubated with 10 mmol/L metformin and/or 10 µmol/L tenovin‐6 for 48 h, and SIRT1 protein levels were measured by immunoblotting. α‐Tubulin was used as a loading control. Results are presented as mean ± SD; *P < 0.05, **P < 0.01

To elucidate the molecular mechanisms underlying the down‐regulation of SIRT1 by metformin in NSCLC cells with different LKB1 statuses, we studied effects of metformin on down‐regulation of SIRT1 in NSCLC cell lines (A549, H1299, H460 and H226). As expected, metformin efficiently down‐regulated SIRT1 expression in functionally LKB1‐negative A549 and H460 cells as well as in H1299 and H226 cells with wild‐type LKB1 in a dose‐dependent manner (Figure 3A). Whether transient knockdown of LKB1 using siRNA could also affect the combination‐induced SIRT1 down‐regulation in H1650 and H1299 cells with wild‐type LKB1 was also explored (Figure 3C,E). Western blot was utilized to investigate knockdown of LKB1 and SIRT1 expression. H1650 and H1299 cells showed suppressed expression of LKB1 protein after treatment with 10 nmol/L or 20 nmol/L siLKB1 (Figure 3B,D). The transient knockdown of LKB1 in H1650 did not affect decreased protein level of SIRT1 caused by metformin and tenovin‐6 as compared with controls (lane 4 vs lane 2; Figure 3C). Similar results were found in H1299 cells (lane 4 vs lane 2; Figure 3E). Additionally, changes of SIRT1 expression by metformin and/or tenovin‐6 after transfection with LKB1^wt^ in LKB1‐deficient A549 cells were investigated. As expected, SIRT1 expression levels were remarkably suppressed in A549 cells with or without LKB1^wt^ by metformin and/or tenovin‐6 treatment as compared with controls (Figure 3F), regardless of their LKB1 status. Interestingly, tenovin‐6 strongly suppressed LKB1 expression in A549 cells with LKB1^wt^. However, inhibition of LKB1 expression by tenovin‐6 did not affect metformin‐mediated SIRT1 reduction. These results suggest that metformin or metformin with tenovin‐6 can effectively down‐regulate SIRT1 expression in NSCLC cells irrespective of LKB1 status.

### 3.4 Metformin and tenovin‐6 synergistically induce p53 acetylation

**Figure 4:**
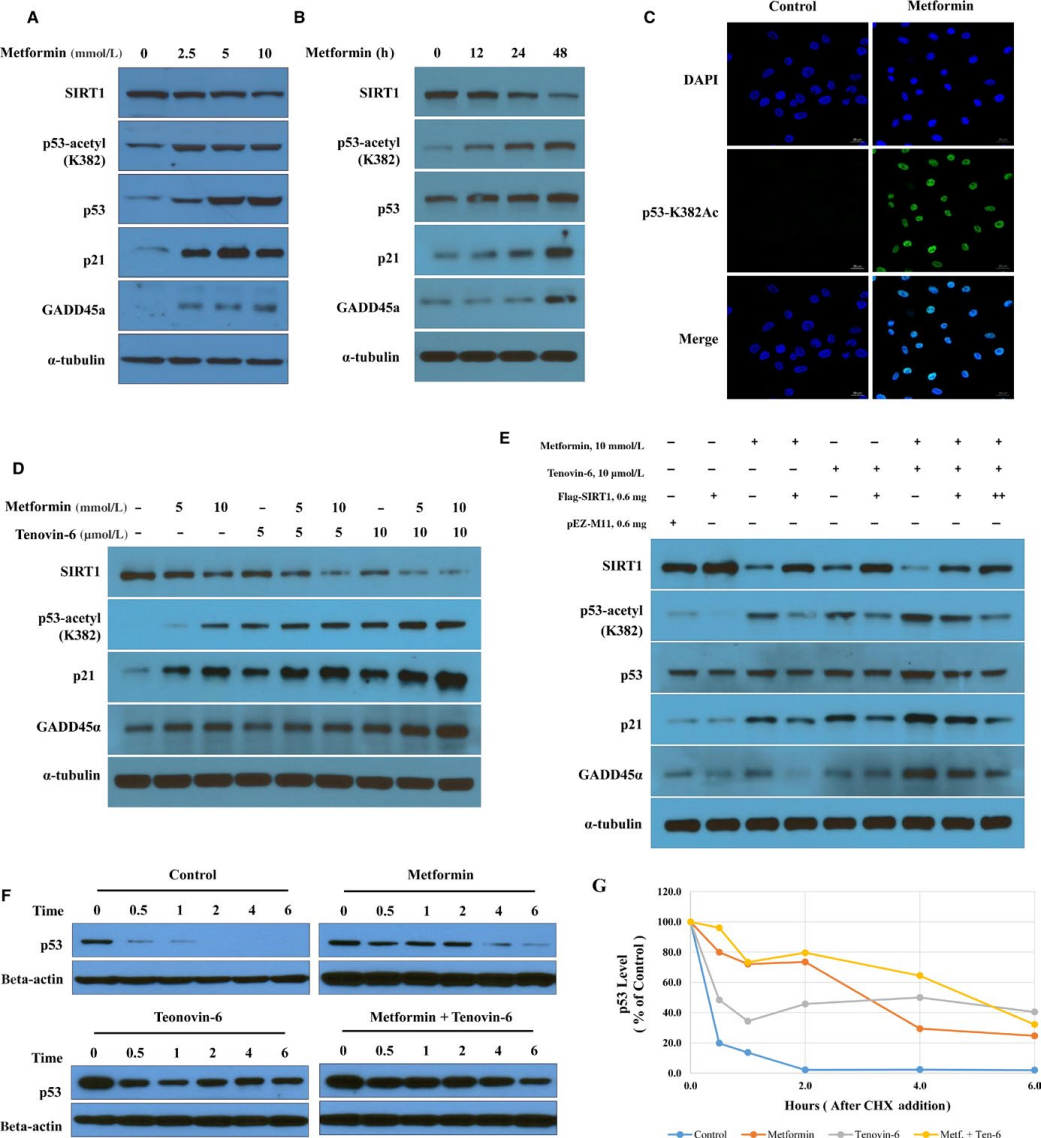
Metformin and tenovin‐6 synergistically inactivate sirtuin 1 (SIRT1) deacetylase activity. (A and B) A549 cells were treated with indicated concentrations of metformin for 48 h (A) and with 10 mmol/L metformin for indicated time (B), then p53 acetylation at K382 and expression levels of p21 and GADD45α were analysed using immunoblotting. (C) The level of acetylated p53 at K382 was measured by immunostaining (green). Scale bars, 20 μm. (D) Acetylation of p53 at K382 and expression levels of p21 and GADD45α in A549 cells treated with different concentrations of metformin and tenovin‐6 for 48 h were analysed. (E) A549 cells were transfected with Flag‐SIRT1 or a vector control (pEZ‐M11) and then treated with metformin alone or in combination with tenovin‐6. Levels of acetylated p53 at K382, total p53, p21 and GADD45α were analysed via immunoblotting at 48 h after treatment. (F) A549 cells were treated with 10 mmol/L metformin or 10 µmol/L tenovin‐6 for 24 h and then incubated with 10 µmol/L Cycloheximide (CHX) for the indicated time. Cell lysates were analysed by immunoblotting using a p53 antibody. (G) To quantify immunoblots from Figure 3F, p53 levels were normalized to β‐actin and results were plotted against the signal obtained at 0 h of CHX treatment

SIRT1 inhibition is known to reduce cell survival through p53 acetylation.^61^ Therefore, this study analysed whether SIRT1 inhibition by metformin and tenovin‐6 could regulate p53 acetylation and downstream target genes. Effects of combination treatment on SIRT1 activity were assessed by examining p53 acetylation at lysine 382, a known SIRT1 deacetylation site. In A549 cells, metformin suppressed SIRT1 expression and induced p53 acetylation at lysine 382. It also increased protein levels of p53, p21 and GADD45α in dose‐ and time‐dependent manners (Figure 4A,B). In addition, immunofluorescent staining intensity of p53 acetylation at lysine 382 was significantly greater in metformin‐treated cells than that in untreated cells (Figure 4C). Combined treatment with metformin and tenovin‐6 also increased p21 and GADD45α expression and p53 acetylation at lysine 382 in A549 cells in a dose‐dependent manner (Figure 4D). Next, this study examined whether the treatment of metformin and/or tenovin‐6 directly act downstream of SIRT1 by testing the effect of SIRT1 overexpression. In addition, p53 acetylation was suppressed by ectopic expression of SIRT1 (lane 2 vs lane 1; Figure 4E). As shown in Figure 4D, metformin and/or tenovin‐6 increased p53 acetylation and p21 and GADD45α levels (lanes 3, 5 and 7 vs lane 1). Increased p53 acetylation and p21 and GADD45α expression were restored by ectopic expression of SIRT1 (lane 7 vs lanes 8 and 9). Our results show that metformin and tenovin‐6 synergistically induced SIRT1 down‐regulation, leading to acetylation (at K382) of p53 and subsequent induction of p21 and GADD45α expression in LKB1‐deficient A549 cells.

To determine whether p53 stability was affected by accumulation of p53 acetylation at lysine 382 and increase in GADD45α protein level, the half‐life of p53 was measured following metformin and/or tenovin‐6 treatment (Figure 4F,G). A549 cells were treated with 10 mmol/L metformin or 10 μmol/L tenovin‐6 for 24 hours and then treated with CHX (25 μg/mL for 0, 0.5, 1, 2, 4 and 6 hours). Metformin and tenovin‐6 alone or in combination substantially increased the half‐life of p53 in A549 cells. Thus, elevated p53 expression (Figure 4A,B,E) may be a result of its increased half‐life. Taken together, these results indicate that combination of metformin with tenovin‐6 can synergistically enhance p53 acetylation and regulate its downstream targets by inhibiting SIRT1 in LKB1‐deficient A549 cells.

### 3.5 Metformin and tenovin‐6 suppress SIRT1 expression by accumulating HIC1 binding to the SIRT1 promoter

**Figure 5:**
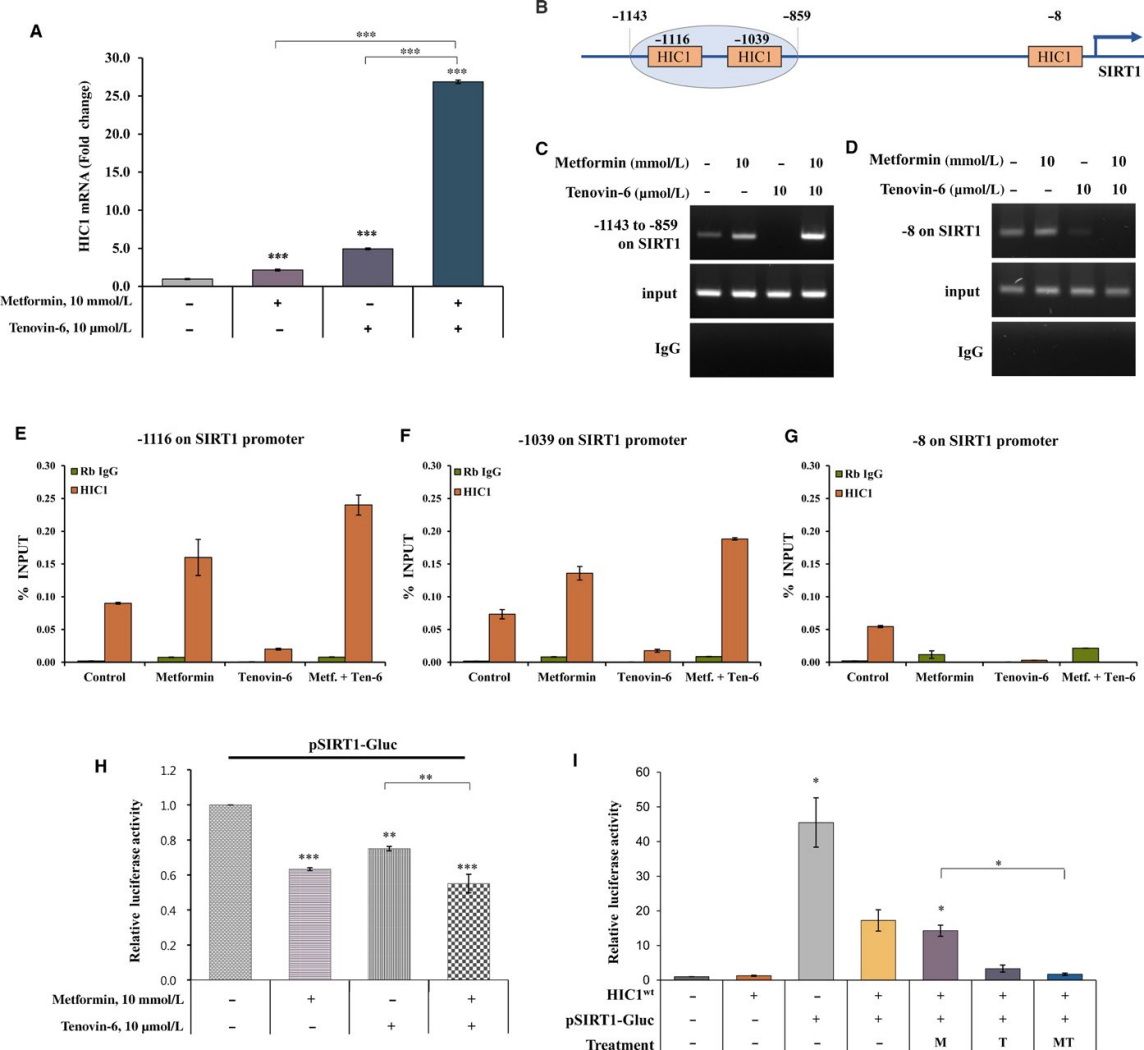
Metformin and tenovin‐6 recruit HIC1 to the SIRT1 promoter. (A) A549 cells were cultured with 10 mmol/L metformin and 10 µmol/L tenovin‐6 alone or in combination for 48 h and HIC1 mRNA levels were measured by qRT‐PCR. Error bars indicate means ± SD; ***P < 0.001 from triplicate experiments. (B) Diagram of the three HIC1 binding sites within the SIRT1 promoter. (C and D) In vitro ChIP assays were conducted in A549 cells treated with 10 mmol/L metformin and 10 µmol/L tenovin‐6 alone or in combination for 48 h. DNA fragments were immunoprecipitated with an anti‐HIC1 antibody and two regions (−1143 to −859 and −8) were amplified using PCR. (E‐G) After immunoprecipitation, HIC1 recruitment to three regions (−1116, −1039 and −8) was analysed by qPCR. Error bars indicate mean ± SD from triplicate experiments. (H) A549 cells were transfected with a SIRT1 promoter plasmid (pSIRT1‐Gluc) containing Gaussia luciferase (Gluc). Transfected cells were cultured in 10 mmol/L metformin and 10 µmol/L tenovin‐6 alone or in combination for 48 h. Luciferase activity was then measured. (I) A549 cells were cotransfected with pSIRT1‐Gluc (or pEZX‐PG02‐Gluc) and plasmids expressing wild‐type HIC1 (HIC1^wt^). Transfected cells were treated with 10 mmol/L metformin and 10 µmol/L tenovin‐6 alone or in combination for 48 h and luciferase activity was measured. “M,” “T” and “MT” indicate metformin (10 mmol/L), tenovin‐6 (10 μmol/L), and a combination of metformin and tenovin‐6 respectively. Experiments shown in H and I were independently performed three times. Data are displayed as mean ± SD; *P < 0.05, **P < 0.01, ***P < 0.001

To understand the possible mechanism underlying SIRT1 down‐regulation by metformin and tenovin‐6, this study analysed the binding of HIC1 to the SIRT1 promoter using chromatin immunoprecipitation. Changes in H1C1 mRNA levels induced by metformin or tenovin‐6 were minimal. However, they were significantly affected by combination treatment with metformin and tenovin‐6 (Figure 5A). There are three HIC1‐binding sites in the human SIRT1 promoter (Figure 5B). HIC1 binding to the SIRT1 promoter at positions −1116 and −1039 bp from the transcription start site was increased substantially after treatment with combination of metformin and tenovin‐6 in comparison with each treatment alone (Figure 5C). After immunoprecipitation, HIC1 recruitment to three regions (−1116, −1039, −8) was measured using qPCR (Figure 5E‐G). ChIP‐qPCR primers were designed to detect more specific regions than ChIP‐PCR primers. Treatment of A549 cells with metformin alone or in combination with tenovin‐6 significantly increased HIC1 recruitment to −1116 and −1039 regions (Figure 5E,F). However, binding was not present at −8 bp upstream (Figure 5D,G). Tenovin‐6 also disturbed the HIC recruitment at only positions −8 bp from the transcription start site of SIRT1 (Figure 5D,G).

To analyse effects of metformin and tenovin‐6 on HIC1 binding to SIRT1 promoter, this study transiently transfected A549 cells with a SIRT1‐luciferase vector (pSIRT1‐Gluc) and then treated them with metformin and/or tenovin‐6 (Figure 5H) or cotransfected them with wild‐type HIC1 (Figure 5I). The combination treatment suppressed SIRT1 transcriptional activity in A549 cells with endogenous HIC1 (Figure 5H). To analyse the effect of HIC1 on SIRT1 transcriptional activity after ectopic expression of wild‐type HIC1, this study transiently cotransfected A549 cells with pSIRT1‐Gluc and HIC1^wt^ followed by treatment with metformin and/or tenovin‐6. SIRT1 luciferase activity in A549 cells with exogenous wild‐type HIC1 was significantly suppressed in response to the combination treatment (Figure 5I). Overall, these results indicated that metformin and tenovin‐6 suppressed SIRT1 transcriptional activity by up‐regulating HIC1 expression, resulting in increased binding of HIC1 to SIRT1 promoter in NSCLC cells. These data also suggest that not all three positions of SIRT1 promoter on the HIC1 recruitment are required for inhibition of SIRT1 promoter activity.

### 3.6 Metformin and tenovin‐6 synergistically promote the apoptotic pathway through SIRT1 down‐regulation in A549 cells

**Figure 6:**
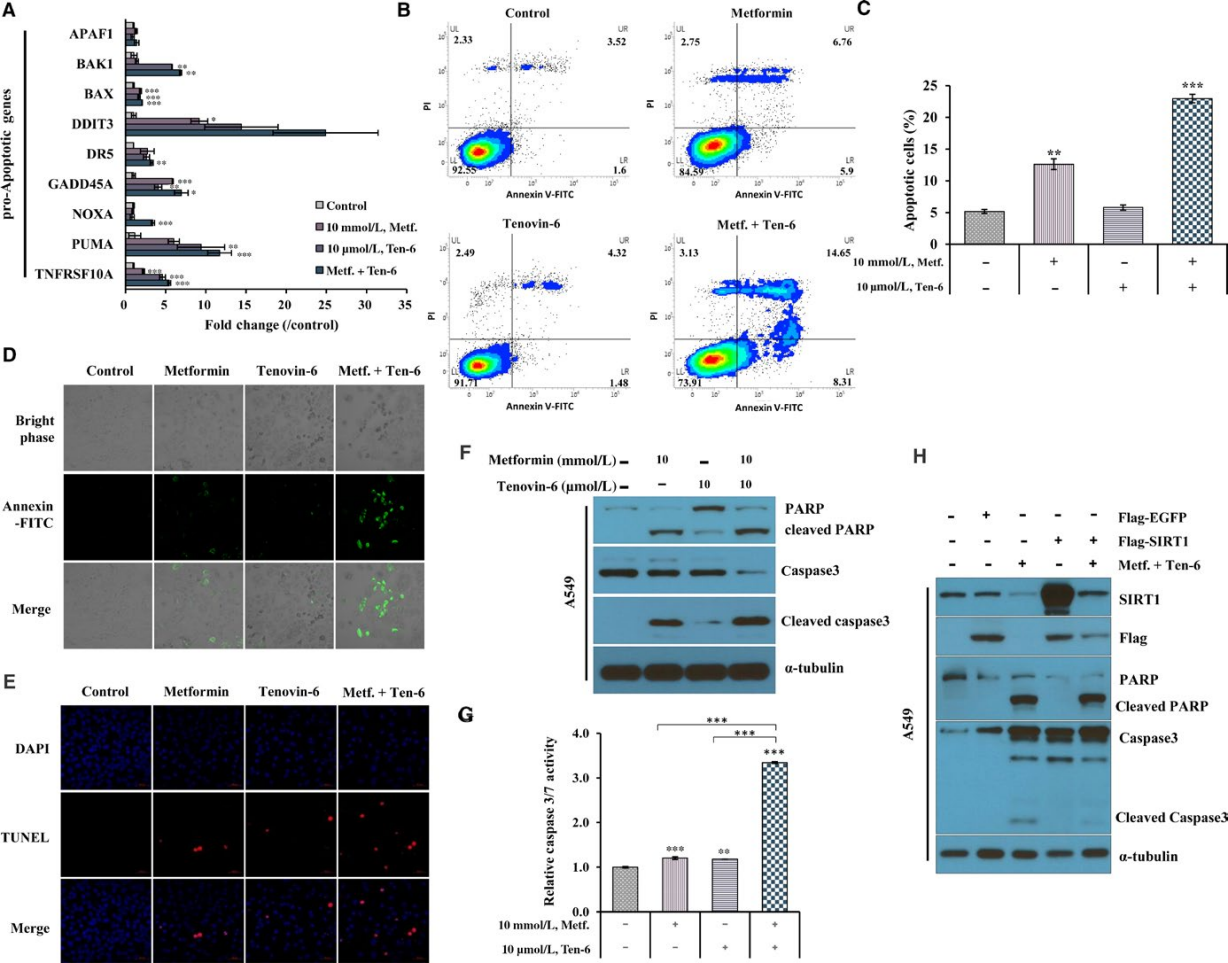
Synergistic effect of metformin and tenovin‐6 on apoptosis. (A) A549 cells were treated with 10 mmol/L metformin and 10 μmol/L tenovin‐6 for 48 h and then mRNA expression levels of pro‐apoptotic genes were measured by qRT‐PCR. Fold change indicates mRNA levels relative to untreated control cells. Error bars indicate mean ± SD from triplicate experiments. (B‐E) For apoptosis assay, A549 cells were treated with 10 mmol/L metformin and 10 μmol/L tenovin‐6 for 48 h. Apoptosis was determined by annexin V‐FITC/PI staining and measured by FACs (B). Apoptotic cells were gated as a percentage of annexin V‐only‐positive cells (C). Experiments shown in A and C were independently performed three times. Data are displayed as mean ± SD; *P < 0.05, **P < 0.01, ***P < 0.001. In addition, apoptotic cells were stained with annexin V conjugated to green fluorescent FITC dye (D) and analysed using a TUNEL assay (E). (F) Immunoblot and analysis of caspase‐3/7 activity. A549 cells were treated with metformin (10 mmol/L) alone or in combination with tenovin‐6 (10 μmol/L) for 48 h. Cell lysates were immunoblotted with antibodies against PARP and Caspase 3 to detect apoptosis. α‐tubulin was used as a loading control. (G) Caspase‐3/7 activity was measured using Caspase‐Glo 3/7 assay kit. Results are displayed as mean ± SD; **P < 0.01, ***P < 0.001. (H) A549 cells were transfected with Flag‐EGFP or Flar‐SIRT1 and treated with or without metformin and tenovin‐6 for 48 h. Cell lysates were then immunoblotted with caspase‐3 and PARP antibodies for activated endogenous caspase‐3/7 activity. Experiments were independently performed three times

SIRT1 is known to repress p53‐dependent transcription, thereby inhibiting p53‐mediated apoptosis following DNA damage or oxidative stress.^62^ This study evaluated the effects of a combination of 10 mmol/L metformin and 10 µmol/L tenovin‐6 on apoptosis of A549 cells. The combination treatment increased mRNA levels of pro‐apoptotic genes such as APAF1, BAK1, BAX, DDIT3, DR5, GADD45α, NOXA, PUMA and TNFRSF10A more effectively than either monotherapy alone in A549 cells (Figure 6A). To determine whether metformin and tenovin‐6 caused cell death by apoptosis, A549 cells were analysed by flow cytometry (FACs) following Annexin V‐FITC and propidium iodide (PI) dual labelling (Figure 6B,C). The percentage of cells that underwent apoptosis as measured by FACs was approximately two times higher in A549 cells treated with metformin than that in control cells (12.68% vs 5.12%, respectively; Figure 6B,C). Apoptosis was weaker for cells treated with tenovin‐6 (5.80%) alone than that for cells treated with metformin (12.68%). However, the combined treatment significantly increased apoptosis (22.96%). In addition, Annexin V staining (Figure 6D) and TUNEL assays (Figure 6E) showed the induction of apoptosis of A549 cells by the combination treatment.

To further confirm apoptosis induction by metformin and tenovin‐6, this study measured cleaved forms of caspase‐3 and PARP (Figure 6F‐6G). Caspase‐3 and PARP were cleaved in the presence of metformin or tenovin‐6 (Figure 6F). However, the combination of metformin and tenovin‐6 induced caspase‐3 activation and PARP cleavage in A549 cells more effectively than either metformin or tenovin‐6 alone. Furthermore, endogenous caspase‐3/7 activity was 3.3 times higher in A549 cells treated with metformin and tenovin‐6 than that in untreated A549 cells (Figure 6G). Overexpression of Flag‐SIRT1 restored the increase in caspase‐3 activation and PARP cleavage (lane 4; Figure 6H). Adding metformin and tenovin‐6 resulted in caspase‐3 activation and PARP cleavage (lane 5; Figure 6H). These results suggest that the combined treatment of metformin and tenovin‐6 can synergistically induce the apoptotic pathway through SIRT1 down‐regulation in A549 cells.

## 4 DISCUSSION

The relationship between SIRT1 overexpression and overall survival of patients with NSCLC has been analysed in several studies. A recent meta‐analysis showed that SIRT1 overexpression was associated with reduced overall survival and that the unfavourable prognostic impact was independent of TNM stage, consistent with our finding.^63^ The absence of an association of SIRT1 expression with pathological score indicates that SIRT1 overexpression occurs from an early stage of NSCLC. Besides, a growing body of evidence suggests that SIRT1 is involved in cancer cell drug resistance through a variety of mechanisms. For example, altered SIRT1 expression in cancer cells contributes in part to cisplatin resistance by altering mitochondrial metabolism. Cisplatin‐resistant cancer cells with high concentrations of NAD^+^ overexpress SIRT1 and show high mitochondrial membrane potential and abnormal mitochondrial ultrastructure.^45^, ^64^ Overexpressed SIRT1 also promotes drug resistance by altering the tumour microenvironment, modifying drug penetration properties of cancer cells, leading to genetic mutations and inducing cancer stem cell‐like properties.^27^ Therefore, targeting SIRT1 in NSCLC patients may provide a novel strategy for improving therapeutic outcome and overcoming cancer drug resistance.

Metformin‐mediated AMPK activation is known to inhibit the mTOR signalling pathway, which controls many biological processes including cell proliferation and cell survival in diverse cancer cell lines. Several studies have shown that the anticancer effect of metformin through the mTOR pathway strictly depends on LKB1 function.^65^, ^66^ LKB1 is a major upstream kinase responsible for AMPK phosphorylation. Loss of LKB1 results in loss of AMPK signalling. Metformin leads to an increase in intracellular ratio of AMP:ATP by disrupting mitochondrial respiration, which in turn leads AMPK activation by LKB1.^29^ Thus, LKB1 and AMPK are critical to metformin's anticancer activity. LKB1‐deficient cells are sensitive to ATP depletion induced by metformin. Besides the mTOR pathway, AMPK is also associated with SIRT1.^67^ It has been suggested that these two proteins have similar effects on diverse processes such as cellular fuel metabolism, inflammation, and mitochondrial function. AMPK and SIRT1 can exert their effects independently or cooperatively by regulating each other.^68^, ^69^ Therefore, this study used NSCLC cells with different LKB1 statuses to understand LKB1/AMPK signalling‐independent effect of metformin on SIRT1. Transient knockdown of LKB1 using siRNA did not affect the combination treatment‐induced SIRT1 down‐regulation in H1650 and H1299 cells with wild‐type LKB1. SIRT1 expression was also inhibited by treatment with metformin and tenovin‐6 in A549 cells that ectopically expressed LKB1, suggesting LKB1/AMPK signalling‐independent effects of metformin and tenovin‐6 on SIRT1.

Interestingly, LKB1 expression in A549 cells with LKB1^wt^ was strongly suppressed after treatment with tenovin‐6 in the present study. Links between the tenovin‐6 and LKB1 expression have not been well studied yet. It has been previously shown that increase in SIRT1 expression can promote deacetylation, ubiquitination and proteosome‐mediated degradation of LKB1 in a senescence model of primary porcine aortic endothelial cells.^70^ However, our results revealed that tenovin‐6 decreased SIRT1 expression in A549 cells with or without ectopic LKB1^wt^. The present study also explored whether the decrease of LKB1 protein expression implied a post‐translational mechanism. The expression of mature miRNAs in A549 cells treated with tenovin‐6 was analysed using a miScript™ miRNA PCR array and expression patterns were analysed (data not shown). miR‐155‐5p was differentially and significantly up‐regulated in A549 cells (fold change = 4.189, relative to that in controls) after treatment with tenovin‐6. Previous studies have shown that miR‐155 targets LKB1 mRNA in glioma cells and cervical cancer cells.^71^, ^72^ We suggest the hypothesis that tenovin‐6‐mediated miR‐155 induction might regulate post‐translational level of LKB1. However, detailed molecular mechanisms underlying the links between tenovin‐6 and miR‐155 remain unclear. Nevertheless, our results might provide some information to guide combinational treatment using metformin and tenovin‐6 to enhance the efficiency of lung cancer treatment regardless of their LKB1 status of patient.

p53 acetylation is known to augment p53 DNA binding, stimulate transactivation of its downstream target genes such as p21 and GADD45α, and regulate p53 stability by inhibiting mouse double minute‐2 (MDM2)‐mediated p53 ubiquitination.^73^ In addition, GADD45α is a conventional downstream gene of p53. It directly participates in the control of cell cycle arrest and apoptosis.^74^, ^75^ However, Jin et al have shown that GADD45α may play a role as an upstream effector in p53 stabilization.^76^ To elucidate the effect of combination treatment on p53 stability, Western blot analysis was performed for GADD45a protein in A549 cells treated with the combination. In this study, SIRT1‐mediated p53 acetylation at Lys382 and induction of GADD45α expression were synergistically increased in response to treatment with both metformin and tenovin‐6. The half‐life of p53 in A549 cells treated with the combination of metformin and tenovin‐6 was prolonged than that in cells without such treatment. These results suggest that the induction of p53 acetylation and GADD45α expression by SIRT1 down‐regulation following combination treatment in LKB1‐deficient A549 cells may help stabilize p53 protein and subsequently induce apoptosis. Besides, SIRT1 down‐regulation caused by the combination treatment was found to be accompanied by caspase3‐dependent apoptosis and p53‐dependent induction of apoptotic genes (such as Noxa, GADD45α, etc.). Interestingly, mRNA levels of GADD45α and DDIT3 were increased greatly in response to treatment with metformin and tenovin‐6 in A549 cells. Both proteins are known to be key regulators of cellular stress response. They stimulate DNA repair and apoptosis. Metformin has inhibitory effect on the production of reactive oxygen species (ROS).^77^ Therefore, it is likely that GADD45α and DDIT3 have an important role in metformin‐mediated reduction in ROS production.

Previous studies have focused on inhibition of the deacetylation activity of SIRT1 by tenovin‐6. Most of these studies confirmed the ability of tenovin‐6 to inhibit SIRT1 activity without showing changes in SIRT1 expression.^45^, ^46^, ^78^, ^79^ In another study, Wei et al showed that the level of SIRT1 was decreased in Omm 1 cells treated with tenovin‐6.^80^ Our study focuses on assessing synergistic effects between metformin and tenovin‐6 in NSCLC cells irrespective of LKB1 status. However, this study showed that tenovin‐6, as well as metformin, suppressed SIRT1 transcriptional activity in A549 cells. Tenovin‐6 was not only involved in the decrease of SIRT1 activity, but also involved in the decrease of SIRT1 expression. The mechanism of tenovin‐6 needs to be determined in further study.

It has been reported that metformin can significantly inhibit tumour cells in vitro at higher concentrations.^81^, ^82^, ^83^ Martin‐Castillo et al have demonstrated that 2 mmol/L of metformin is at least 50‐fold excess over plasma concentration in patients.^84^ However, Carvalho et al have shown that metformin accumulates in tissues at concentrations several fold higher than those in blood.^85^ This indicates that concentrations of metformin similar to those used in preclinical models (1‐10 mmol/L) might be attained during cancer treatment. In addition, Morgillo et al have shown that the positive charge of metformin could promote its accumulation within the mitochondrial matrix (> 20 mmol/L).^86^ Thus, the dosage of metformin using in vitro remains controversial. Nevertheless, metformin as clinically approved drug is more attractive for treatment of tumour cells. To overcome this problem, this study investigated whether a combination treatment at lower concentrations instead of concentrations of each single agent commonly used in vitro could have synergistic effects. IC_50_ values of metformin and tenovin‐6 were 28.7 mmol/L and 21.1 μmol/L, respectively (data not shown). However, we used low concentrations of metformin and tenovin‐6. Our results reveal synergistic effects to regulate SIRT1 expression by the combination of metformin with tenovin‐6.

A serious problem in treating lung cancer is that some patients continue to smoke even after their diagnoses. Continuous exposure to tobacco smoke may influence the efficacy of chemotherapeutic agents.^87^ Therefore, in vitro studies with or without exposure to NNK (Nicotine‐derived nitrosamine ketone) were performed. A549 cells were incubated with NNK for 2 days and then with metformin for 2 days. Metformin decreased both expression and phosphorylation of SIRT1 but increased p53 acetylation in a time‐dependent manner (data not shown), suggesting that metformin might be effective even in smokers.

In summary, this study reveals that SIRT1 overexpression is associated with poor survival in NSCLC patients. This study also provides a mechanism for antitumour effects of targeting SIRT1 in NSCLCs. Results of this study showed that combination of metformin and tenovin‐6 acted synergistically in inhibiting cell growth in NSCLC cells irrespective of LKB1 status through inhibition of SIRT1 expression. Metformin with tenovin‐6 synergistically down‐regulated SIRT1 expression by recruiting HIC1 on the SIRT1 promoter; subsequently, this resulted in accumulation of p53 acetylation and induction of the apoptotic pathway in a functional LKB1‐deficient NSCLC cells. Moreover, the synergy between the combination of low doses of metformin and tenovin‐6 exerted antitumour effects in NSCLC cells. These data suggest that the combination of metformin and tenovin‐6 could enhance antitumour effects through LKB1‐independent SIRT1 down‐regulation in NSCLCs. This consideration opens new possibilities for combination of metformin with SIRT1 inhibitors in NSCLC cells irrespective of LKB1 status.

## CONFLICT OF INTEREST

The authors declare no conflict of interest.

## AUTHOR CONTRIBUTIONS

BBL and DHK designed the overall study and drafted the manuscript. BBL performed the experimental work and data analysis in vitro. YK and DK contributed to immunohistochemistry. EYC, HH and DHK performed data interpretation and data analyses of immunohistochemical staining. All surgeries for patients were performed by HKK and YMS. All authors read and approved the final manuscript.
